# Study on the Structural Effect of Maltoligosaccharides on Cytochrome c Complexes Stabilities by Native Mass Spectrometry

**DOI:** 10.1007/s13659-017-0150-x

**Published:** 2018-01-29

**Authors:** Quan Chi, Ying-Zhi Liu, Xian Wang

**Affiliations:** Key Laboratory of Analytical Chemistry of the State Ethnic Affairs Commission, College of Chemistry and Materials Science, South-Central University for Nationalities, Wuhan, Hubei 430074 People’s Republic of China

**Keywords:** Electrospray ionization mass spectrometry, Maltoligosaccharides, Cytochrome c complexes, Structure-binding relationship

## Abstract

**Abstract:**

Noncovalent interactions between ligands and targeting proteins are essential for understanding molecular mechanisms of proteins. In this work, we investigated the interaction of Cytochrome c (Cyt c) with maltoligosaccharides, namely maltose (Mal II), maltotriose (Mal III), maltotetraose (Mal IV), maltopentaose (Mal V), maltohexaose (Mal VI) and maltoheptaose (Mal VII). Using electrospray ionization mass spetrometry (ESI–MS) assay, the 1:1 and 1:2 complexes formed by Cyt c with maltoligosaccharide ligand were observed. The corresponding association constants were calculated according to the deconvoluted spectra. The order of the relative binding affinities of the selected oligosaccharides with Cyt c were as Mal III > Mal IV > Mal II > Mal V > Mal VI > Mal VII. The results indicated that the stability of noncovalent protein complexes was intimately correlated to the molecular structure of bound ligand. The relevant functional groups that could form H-bonds, electrostatic or hydrophobic forces with protein’s amino residues played an important role for the stability of protein complexes. In addition, the steric structure of ligand was also critical for an appropriate interaction with the binding pocket of proteins.

**Graphical Abstract:**

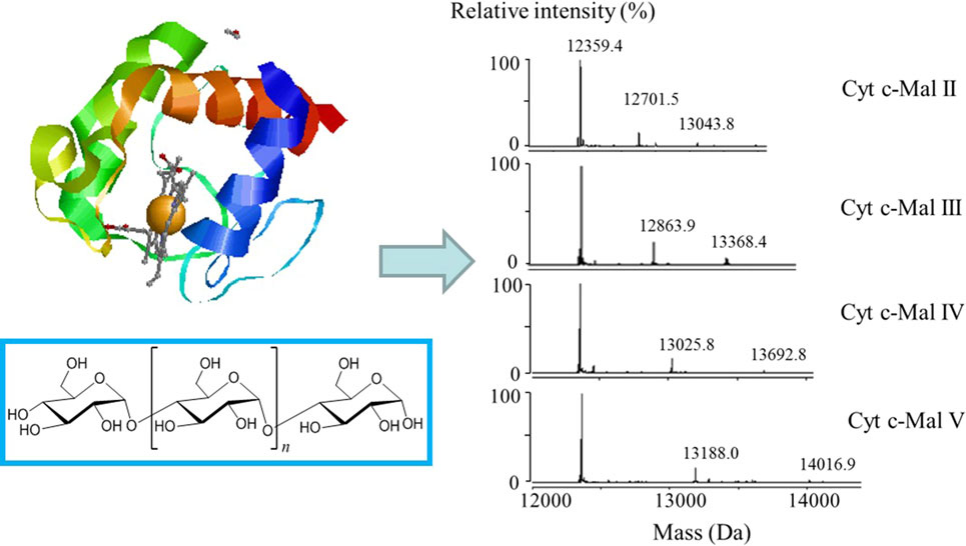

## Introduction

Noncovalent complexes formed by biological macromolecules and pharmceutical molecules are important in biological and biomedical study [1, 2]. Native mass spectrometry using electrospray ionization mass spetrometry (ESI–MS) has proven to be a powerful tool in the study of noncovalent interactions [3, 4], and the investigation of noncovalent protein complexes in solution with ESI–MS has been successfully demonstrated [5–11] and reviewed [12–15]. Electrospray is a soft ionization technique that introduces the weakly bound complexes formed in solution into the gas phase where they can be analyzed by mass spectrometry [12]. For years, ESI–MS has been used to reveal the existence of noncovalent protein complexes, and to provide stoichiometric information and association/dissociation constants [5, 8, 16, 17]. The binding constants measured by ESI–MS have been found to correlate well with those measured by solution based techniques. Various types of noncovalent complexes were studied by ESI–MS, such as protein-organic ligand [9, 17, 18], protein-metal [19–21], protein–sugar [5, 8, 22, 23] and protein–protein [24] complexes.

Cytochrome c (Cyt c) is a small hemeprotein associated with the inner membrane of the mitochondrion. Unlike other cytochromes, Cyt c is an essential component of the electron transport chain, where it carries one electron [21]. It is highly water-soluble, and is easy to ionize and stable in a wide pH range. Therefore, Cyt c was selected as a model protein in this work, in order to study the main effects on the stability of noncovalent protein complexes. In our previous work, we have investigated the interactions between Cyt c and saikosaponins by ESI–MS [10] and found that the glycosyls were the key functional groups rather than their triterpene moieties. The structure differences of the glycosyl moities have shown to have little effect on the binding activity with Cyt c. Furthermore, we have found that hydrogen bonds play a significant role in the formation of flavonoid or isoflavonoid bound Cyt c complexes [9].

**Fig. 1:**
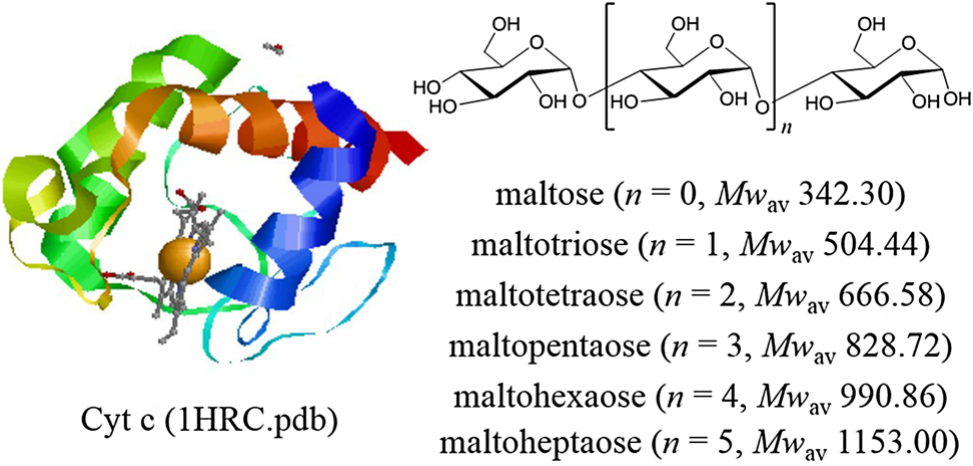
Structures of Cyt c of a ribbon diagram were adapted from 1HRC.pdb [1] and maltooligosaccharides. The diagram was created using the RasMol program [2]

This work further explored the effect of steric structure and hydroxyl numbers in ligands on the binding affinities of ligands, by studying the Cyt c-maltooligosaccharide complexes. Maltooligosaccharides are oligosaccharides which are composed of two to ten glucoses connected by *α*-(1→4)-glucosides. Six homologue maltooligosaccharides (Mal) were chosen as the ligands binding to Cyt c, namely, maltose (Mal II) maltotriose (Mal III), maltotetraose (Mal IV), maltopentaose (Mal V), maltohexaose (Mal VI), maltoheptaose (Mal VII). The structures of these ligands and Cyt c are shown in Fig. 1 [25, 26].

## Results and Discussion

### Observation of the Noncovalent Cyt c Complexes with Maltooligosaccharides by ESI–MS

**Fig. 2:**
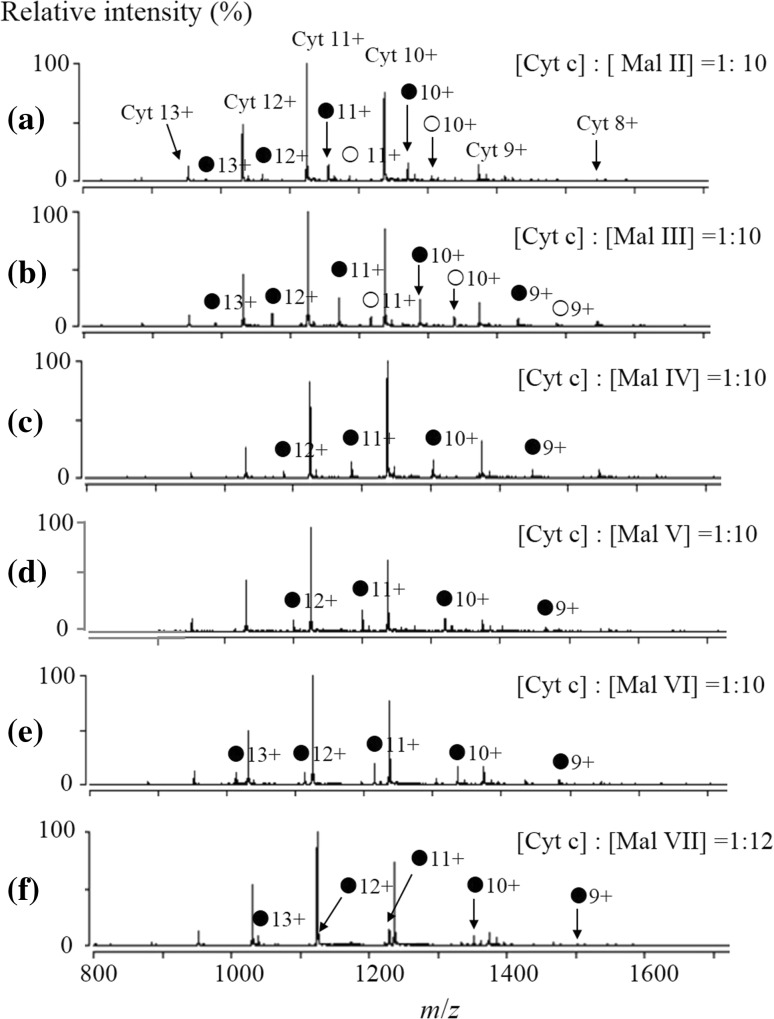
ESI mass spectra for the complexes of cytochrome c with maltose (**a**), maltotriose (**b**), maltotetraose (**c**), maltopentaose (**d**), maltohexaose (**e**), maltoheptaose (**f**).  represents the 1:1 complexes,  represents the 1:2 complexes. The concentration ratio between Cyt and maltooligosaccharides is 1:10 ([Cyt c]:[Mal VII] = 1:12), while the concentration of Cyt is 0.50 μmol L^−1^)

**Fig. 3:**
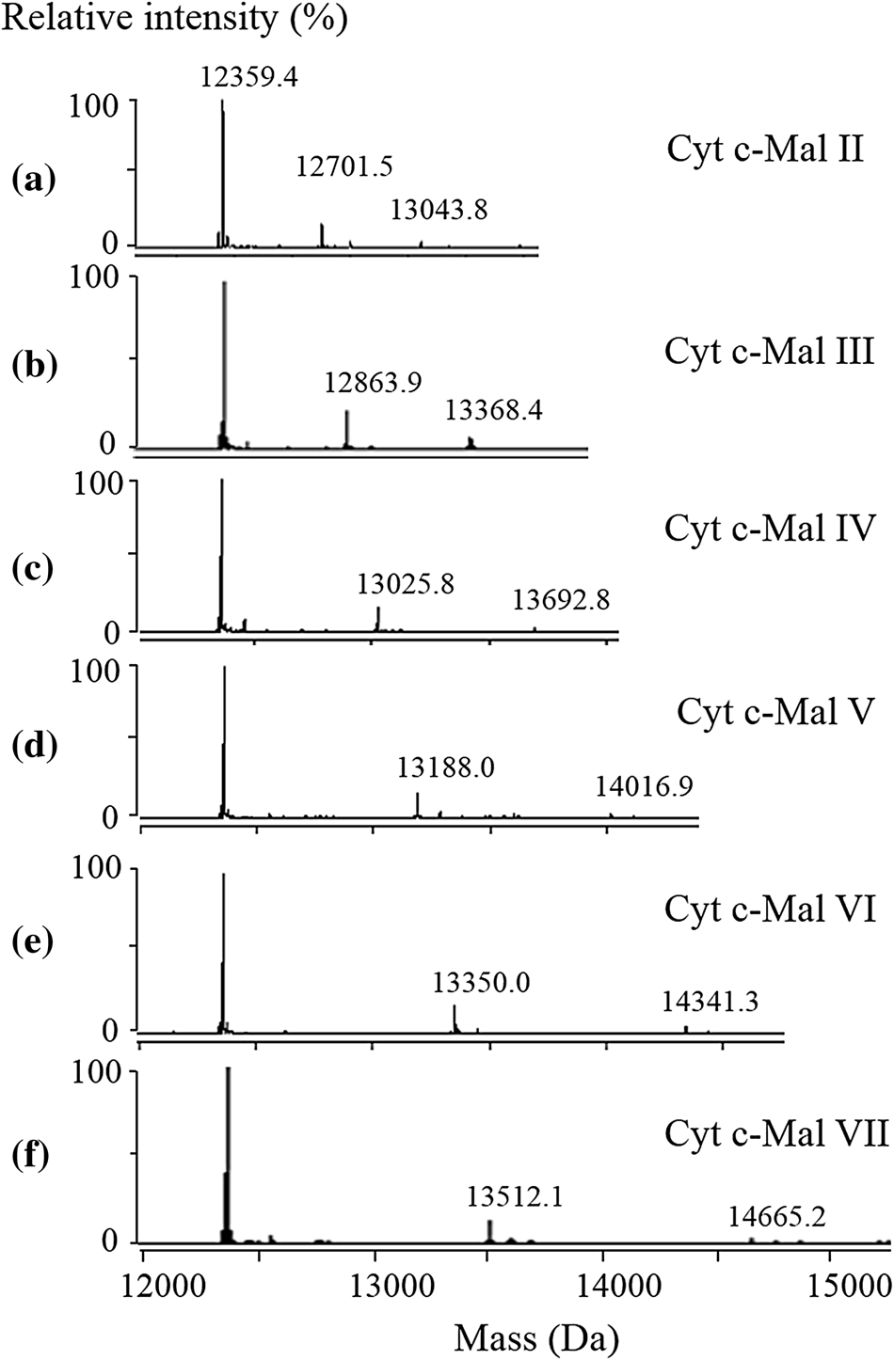
Deconvoluted spectra for the complexes of cytochrome c with maltose (**a**), maltotriose (**b**), maltotetraose (**c**), maltopentaose (**d**), maltohexaose (**e**), maltoheptaose (**f**). The concentration ratio between Cyt and maltooligosaccharides is 1:10 ([Cyt c]:[Mal VII] = 1:12), while the concentration of Cyt is 0.50 μmol L^−1^)

ESI–MS spectrum for the aqueous solution of Cyt c (0.50 μM) and Mal II (5 μM) is shown in Fig. 2a. The mass spectrum displays abundant ions corresponding to Cyt c and the Mal II-bound complexes with a charge distribution of 8 + to 13 +. The deconvoluted spectrum (Fig. 3a) provides directly the binding stoichiometry of the formed Cyt c complexes. The 1:1 and 1:2 Cyt c-Mal II complexes were consistently detected with comparable relative abundance at different concentration ratios of Cyt c-Mal II solutions. The measured molecular mass of Cyt c from equine heart was 12359.4 Da. The 1:1 and 1:2 Cyt c-Mal II complexes arose the peaks of 12701.5 and 13043.8 Da in the deconvoluted mass spectra, respectively.

Previous study has verified that the binding of Cyt c with Mal II did not arise from the nonspecific interaction during the ionization in the ESI–MS [10]. Therefore, its homologous maltooligosaccharides shared the same phenomena. Cyt c-Mal complexes observed in ESI–MS reflected the corresponding species present in solution.

The formation of Cyt c complexes with other maltooligosaccharides, from Mal III to Mal VII, were also observed. The ESI and deconvoluted mass spectra for each complex are shown in Figs. 2b–f and 3b–f. Ions of free Cyt c and noncovalent complexes were observed in their ESI mass spectra, predominantly at the charge states of 8 + to 13 +. The formed 1:1 and 1:2 protein–ligand complexes were easily distinguished based on the measured molecular weights in deconvoluted mass spectra. For example, in Fig. 2b, the peak of 12359.4 Da is corresponding to the molecular weight of free Cyt c, and the peak of 12863.9 Da can be assigned to the 1:1 complex of Cyt c and Mal III with the molecular weight of 504.5 Da. The peak of 13368.4 Da is assigned to the 1:2 complexes of Cyt c and Mal III.

### Determination of Association Constants of Cyt c-Maltooligosaccharide Complexes

Based on the equations given in experimental Sect. “Calculation of Association Constants”, the stepwise association constants *K*_a,1_ and *K*_a,2_ values of Cyt c-maltooligosaccharide complexes were calculated according to the corresponding peak ratios in deconvoluted mass spectra (Fig. 3), and shown in Table 1. The relative standard deviation (RSD) values of *K*_a,1_ at five different Cyt c-ligand concentration ratios (i.e., 1:6, 1:8, 1:10, 1:12 and 1:14) are usually < 3.0% (Table 1). The RSD values of *K*_a,2_ are higher than those of *K*_a,1_ because the ion intensities of PL_2_ complexes are much lower than those of PL_1_. By comparing the *K*_a,1_ values of these homologue ligand-bound Cyt c, the order of the binding affinity of these maltooligosaccharides binding to Cyt c has determined to be: Mal III > Mal IV > Mal II > Mal V > Mal VI > Mal VII.

**Table 1:** The calculated association constants of the cytochrome c complexes with maltooligosaccharides

Complexes	*K*_a,1_ (10^4^ L mol^−1^)	RSD (%, *n* = 15)	*K*_a,2_ (10^4^ L mol^−1^)	RSD(%, *n* = 15)
Cyt c-Mal II	3.227	2.9	4.278	6.3
Cyt c-Mal III	3.842	2.5	6.826	15
Cyt c-Mal IV	3.279	5.6	4.480	4.0
Cyt c-Mal V	2.654	0.80	3.261	4.2
Cyt c-Mal VI	2.438	2.5	3.591	8.4
Cyt c-Mal VII	2.205	1.6	3.932	11

Ligand structure is the intrinsic factor that affects the stability of protein complex. Previous work has found that H-bonds are the main interaction force for the formation of noncovalent Cyt c complexes with flavonoid compounds whose structure contains multiple-hydroxyl functional groups [9]. Theoretically, when there are more H-bond donor groups in the ligands, more H-bonds will be formed between ligands and interacting amino acid residues of protein, so the binding affinity of the ligand should be stronger. However, the binding affinity of Mal II to Mal VII with Cyt c does not increase with the increasing numbers of –OH groups according to the above results. Mal III has the highest binding affinity, whereas the binding affinity of Mal II and Mal IV are nearly identical and the binding affinity declines from Mal V to Mal VII. Obviously, the molecular size from Mal II to Mal VII with linear structures become larger with increasing molecular weights. The steric hindrance may prevent Mal ligands of larger sizes from approaching the interacting amino acid residues in the binding pocket of protein. Hence, there are two major structural aspects affect the stability of the interaction between protein and small ligands. One is the hydroxyl functional groups of ligands and the other one is the steric structure of ligands. The former aspect determines the types of interacting forces. The latter selects the ligand with appropriate size that can well fit the binding pocket of protein. In the present study, Mal III has the highest affinity with Cyt c because of its suitable size and relatively more –OH groups than its other homologues.

## Experimental Section

### Materials and Sample Preparation

Cyt c from horse heart (purity ≥ 95%) was obtained commercially as lyophilized powder from Sigma-Aldrich (St Louis, MO, USA) and was used without further purification. Maltose (purity ≥ 96%) was obtained from Shanghai Xinran Biotechnology Co., Ltd. (Shanghai, China). Maltotriose, maltotetraose, maltopentaose, maltohexaose and maltohepose (purity ≥ 98%) were also obtained from Sigma-Aldrich (St Louis, MO, USA). HPLC-grade methanol was purchased from Fisher Scientific (Waltham, MA, USA). Glacial acetic acid was purchased from Sinopharm Chemical Reagent Co. Ltd. (Shanghai, China). The water purification system was obtained from Mole Scientific Instrument Co., Ltd (Shanghai, China).

Cyt c was mixed with appropriate amounts of stock solutions of ligands to a final concentration of 0.5 μmol L^−1^ with different concentrations of ligands at 3, 4, 5, 6 and 7 μmol L^−1^, respectively, giving the molar concentration ratios of 1:6, 1:8, 1:10, 1:12 and 1:14. The incubation time for each mixture at room temperature was 1 h. Before ESI–MS analysis the solutions were centrifuged at 5000 r min^−1^ for 1 min.

### ESI–MS

All mass spectra were acquired from an Agilent LC–MS system (an Agilent 1200 series HPLC coupled to an Agilent 6520 ESI quadrupole time-of-flight mass spectrometer with a standard ESI source) in the positive ion mode. The samples were injected via a syringe pump introduced into the ESI source at a flow rate of 6 μL min^−1^. The Cyt c-Mal II complex was served as a model ligand-bound Cyt c for establishing the appropriate instrumental conditions for the ESI–MS measurements. The ESI source parameters were optimized in a stepwise fashion to achieve a stable complexes signal and to minimize the gas-phase decomposition of the noncovalent complexes. The gas temperature was 130 °C. The flow of drying gas was 8 L min^−1^, and the nebulizer was at 15 psi. The voltage of the capillary was 3500 V, and the fragmentor was 125 V. Each experiment was repeated in triplicate.

### Calculation of Association Constants

The stepwise association constants *K*_a,*i*_ of protein–ligand complex (PL_*n*_) can be determined by using the direct ESI–MS assay [17], based on quantification of the relative intensities of ligand-bound protein complexes and free protein in the ESI mass spectra, namely *I*_PL*n*_ and *I*_P_. ESI–MS studies have shown that when the ligand is small compared to the protein, the size and surface properties of the free and ligand-bound proteins are similar, the magnitude of ion relative intensity detected in the gas phase is representative of the magnitude of the equilibrium concentration of the corresponding species in solution [5, 8, 17]. Then the distribution coefficient (*r*) of free protein (P) or ligand-bound protein (PL_*i*_) in solution can be measured by the relative peak intensity (*I*_P_ or *I*_PL*i*_) ratio in the ESI mass spectra, which expressed as follow equations.

1 $$\begin{aligned} r_{0} = \frac{{ [ {\text{P}}]}}{{ [ {\text{P]}}_{0} }} = \frac{{ [ {\text{P}}]}}{{ [ {\text{P]}} + [ {\text{PL}}] + [ {\text{PL}}_{2} ] + \cdot \cdot \cdot + [ {\text{PL}}_{n} ]}} \hfill \\ \;\;\; = \frac{{I_{\text{P}} }}{{ \, I_{\text{P}} + I_{\text{PL}} + I_{{{\text{PL}}_{2} }} + \cdot \cdot \cdot + I_{{{\text{PL}}_{n} }} }} = \frac{{I_{\text{P}} }}{{ \, I_{\text{P}} + \sum\nolimits_{i = 1}^{n} {I_{{{\text{PL}}_{i} }} } }} \hfill \\ \end{aligned}$$

2 $$r_{i} = \frac{{ [ {\text{PL}}_{i} ]}}{{ [ {\text{P]}}_{0} }} = \frac{{I_{{{\text{PL}}_{i} }} }}{{ \, I_{\text{P}} + \sum\nolimits_{i = 1}^{n} {I_{{{\text{PL}}_{i} }} } }}$$

Here, [P]_0_ is the analytical concentration of protein in solution, and calculation of the association constants *K*_a,*i*_ are deduced briefly as follows.

3 $$\left[ {\text{P}} \right] \, = r_{0} \left[ {\text{P}} \right]_{0}$$

4 $$\left[ {{\text{PL}}_{i} } \right] \, = r_{i} \left[ {\text{P}} \right]_{0}$$

5 $$\begin{aligned} \left[ {{\text{P}}\left] { \, = \, } \right[{\text{P}}} \right]_{0} - \, \left[ {{\text{PL}}\left] { \, - \, } \right[{\text{PL}}_{ 2} } \right] \, - \cdot \cdot \cdot - \, \left[ {{\text{PL}}_{n} } \right] \hfill \\ = \, \left[ {\text{P}} \right]_{0} - r_{ 1} \left[ {{\text{P}}\left] {_{0} - r_{ 2} } \right[{\text{P}}} \right]_{0} - \cdot \cdot \cdot - r_{n} \left[ {\text{P}} \right]_{0} \hfill \\ \end{aligned}$$

6 $$\begin{aligned} \left[ {{\text{L}}\left] { \, = \, } \right[{\text{L}}} \right]_{0} - \, \left[ {{\text{PL}}\left] { \, - { 2}} \right[{\text{PL}}_{ 2} } \right] \, - \cdot \cdot \cdot - n\left[ {{\text{PL}}_{n} } \right] \hfill \\ = \, \left[ {\text{L}} \right]_{0} - r_{ 1} \left[ {{\text{P}}\left] {_{0} - { 2}r_{ 2} } \right[{\text{P}}} \right]_{0} - \cdot \cdot \cdot - nr_{n} \left[ {\text{P}} \right]_{0} \hfill \\ \end{aligned}$$

So the stepwise association constants can be calculated by the follow equations.

7 $$K_{\text{a,1}} = \frac{{\left[ {\text{PL}} \right]}}{{\begin{array}{*{20}c} {\left[ {\text{P}} \right]} & {\left[ {\text{L}} \right]} \\ \end{array} }} = \frac{{r_{1} }}{{r_{0} \left( {\left[ {\text{L}} \right]_{0} - \left[ {\text{P}} \right]_{0} \left( {r_{1} + 2r_{2} + \cdots + nr_{n} } \right)} \right)}} = \frac{{r_{1} }}{{r_{0} \left( {\left[ {\text{L}} \right]_{0} - \left[ {\text{P}} \right]_{0} \sum\nolimits_{i = 1}^{n} {ir_{i} } } \right)}}$$

8 $$K_{{{\text{a,}}i}} = \frac{{[{\text{PL}}_{i} ]}}{{[{\text{PL}}_{i - 1} ][L]}} = \frac{{r_{i} }}{{r_{i - 1} \left( {[{\text{L}}]_{0} - [{\text{P}}]_{0} \sum\nolimits_{i = 1}^{n} i r_{i} } \right)}}$$

## Conclusion

In this work, the interactions of Cyt c and a series of maltooligosaccharide ligands were characterized using native mass spectrometry method. The 1:1 and 1:2 Cyt c-Mal complexes were observed by ESI–MS, and the stepwise association constants were calculated according to the deconvoluted spectra. The order of the relative binding affinities of the selected oligosaccharides with Cyt c were as Mal III > Mal IV > Mal II > Mal V > Mal VI > Mal VII. The results indicated that the stability of noncovalent protein complexes was correlated to the molecular structure of ligands, including their functional groups and steric structures. Such study of the structure-binding relationship of noncovalent protein complexes would help for the drug screening and de novo therapeutic agent design.
